# Selective bacterial separation of critical metals: towards a sustainable method for recycling lithium ion batteries†

**DOI:** 10.1039/d2gc02450k

**Published:** 2022-10-17

**Authors:** Virginia Echavarri-Bravo, Houari Amari, Jennifer Hartley, Giovanni Maddalena, Caroline Kirk, Maarten W. Tuijtel, Nigel D. Browning, Louise E. Horsfall

**Affiliations:** School of Biological Sciences, University of Edinburgh Edinburgh EH9 3FF UK Louise.Horsfall@ed.ac.uk; Department of Mechanical, Materials and Aerospace Engineering, University of Liverpool Liverpool L69 3GQ UK; School of Chemistry, University of Leicester Leicester LE1 7RH UK; School of Chemistry, University of Edinburgh Edinburgh EH9 3FJ UK; Faraday Institution (ReLiB project) Quad One Harwell Science and Innovation Campus Didcot UK; Sivananthan Laboratories 590 Territorial Drive Bolingbrook IL 60440 USA; Physical and Computational Sciences Directorate, Pacific Northwest National Laboratory P.O. Box 999 Richland WA 99352 USA

## Abstract

The large scale recycling of lithium ion batteries (LIBs) is essential to satisfy global demands for the raw materials required to implement this technology as part of a clean energy strategy. However, despite what is rapidly becoming a critical need, an efficient and sustainable recycling process for LIBs has yet to be developed. Biological reactions occur with great selectivity under mild conditions, offering new avenues for the implementation of more environmentally sustainable processes. Here, we demonstrate a sequential process employing two bacterial species to recover Mn, Co and Ni, from vehicular LIBs through the biosynthesis of metallic nanoparticles, whilst Li remains within the leachate. Moreover the feasibility of Mn recovery from polymetallic solutions was demonstrated at semi-pilot scale in a 30 L bioreactor. Additionally, to provide insight into the biological process occurring, we investigated selectivity between Co and Ni using proteomics to identify the biological response and confirm the potential of a bio-based method to separate these two essential metals. Our approach determines the principles and first steps of a practical bio-separation and recovery system, underlining the relevance of harnessing biological specificity for recycling and up-cycling critical materials.

## Abstract 2

Harnessing bacterial processes for the selective separation of strategic and critical metals contained in spent lithium-ion batteries (LIBs).

## Graphical abstract

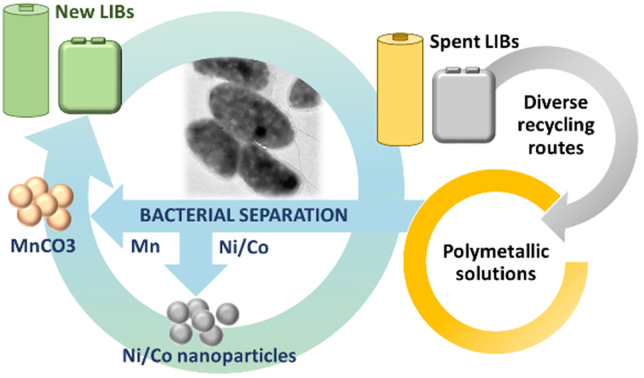

## Introduction

It is well established that one of the most important measures to slow climate change is a reduction in CO_2_ emissions. Road transportation is highly dependent on carbon-based fuels and responsible for 20% of CO_2_ emissions worldwide.^1^ Therefore, there is mounting pressure to move towards transportation alternatives with lower carbon footprints, leading to many national governments incentivising the transition to electric vehicles (EV) for mobility. These pressures and incentives are resulting in an increasing demand for lithium ion batteries (LIBs),^2^ currently the best technological solution to power EV based on energy density.^3^ Life cycle assessment of LIBs shows that the availability of raw materials needed to fulfil the demand for EV LIBs by 2050 is estimated to be ‘very critical’ for both lithium and cobalt, and ‘critical’ for nickel.^4,5^ Thus, the development of efficient technologies to enable selective recovery and recycling of the components and materials present in spent LIBs is vital for minimising risks in the supply chain and reducing the waste burden.^6^ Moreover, moving to a circular economy for LIBs would reduce reliance on the current sources of raw materials associated with human rights abuses and decrease mining activities reported to negatively impact upon human and environmental health.^7,8^

Adaptive and flexible recycling solutions are needed to address the wide variety of continuously evolving cathode chemistries in a highly competitive market. Current recycling methods are multi-step processes, often starting with physical separation of the various battery parts, or shredding and comminution, followed by a combination of other physical, hydrometallurgical and/or pyrometallurgical processes.^3,6,7^ Pyrolysis involves the calcination (>400 °C) of the battery and although it is a mature technology it has been decreasing in popularity for EV LIB recycling. It results in high CO_2_ and toxic emissions, and the significant loss of value to the materials streams including Li and Al.^7^ Hydrometallurgical methods involve the dissolution of battery parts, often using sulfuric acid or hydroxides,^9^ with other secondary treatments (*e.g.* thermal, sonication) that result in the production of battery leachates with varying concentrations of metals dependent on the chemistry of the cathode. Development of more efficient recycling options continues at all stages of the recycling loop, with new technologies investigated, such as high powered ultrasound with increasing potential for the rapid delamination of electrodes and to facilitate direct recycling of the cathode.^10^ Nevertheless, hydrometallurgy is always being applied to some extent and polymetallic solutions that require metal separation and refining are generated.

As such, this too is an evolving area of study and novel solutions have been reported such as ferro-chemistry-based approaches and bio-electrochemical reduction to separate Li from Co.^11,12^ However when other elements are also present in the cathode, such as Mn and Ni (*e.g.* LiNi_*x*_Co_*y*_Mn_*z*_O_2_, NCM) the downstream recovery and separation of metals contained in the battery leachates is commonly achieved by solvent-exchange (SX) and chemical precipitation. The energy inputs and the use of hazardous chemicals are then major limiting factors for achieving a cost effective and sustainable process.^3,7^ SX methods using organophosphorus solvents are the most frequent approach to selectively separate and purify metals, with D2EHPA being the most common method for Mn separation and Cyanex®272 for Co and Ni. However SX involves the use of toxic solvents to treat aqueous polymetallic streams^13^ and Mn removal using SX is not cost-efficient unless subsidised by the recovery of Co and Ni.^14^ Other limitations of SX include the treatment of heterogeneous feedstocks that may hinder the control of the process; the potential environmental impacts of organic solvents if the reuse rates are low, and the wastewater generated.^14^ There are other chemical alternatives to SX which may first involve the selective leaching of Li using organic acids (*e.g.* tartaric, formic, oxalic) followed by precipitation as Li_2_CO_3_ from the addition of NaOH and Na_2_CO_3_.^15,16^ The remaining metals in the cathode once dissolved in inexpensive acids (*e.g.* H_2_SO_4_) are precipitated as hydroxides^17^ or carbonates^15^ to be used as precursor material for new active electrodes. The high requirements of alkaline compounds (*e.g.* NaOH, NH_4_OH, Na_2_CO_3_) with an elevated environmental footprint is one of the major drawbacks of the chemical precipitation approach.^18^ The energy inputs of the chemical reactions vary within a wide range, as solvent extraction takes place ∼25 °C, while chemical precipitation as hydroxides or carbonates may require 40–65 °C^15–17^ or above (80 °C) to shorten reaction times, enhance metal specificity and evaporate the solvent. Multiple combinations of optimised parameters are reported in the literature dependent upon the initial concentration of metals, leaching process, target efficiency and purity.^19^ Nowadays the development of greener methods that enable the selective separation of metals from battery leachates and allows for their return to use is a major challenge for the industry.^3^

**Scheme 1:**
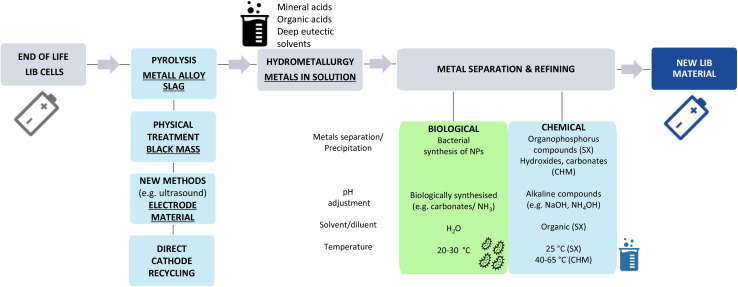
Recycling scheme depicting conventional and novel recycling processes for end of life LIB cells and resulting fractions (underlined) containing metals. The reaction conditions of the metal bioseparation process presented in this study (green box) is depicted side by side with conventional chemical separation methods (SX: solvent exchange; CHM: chemical precipitation with *e.g.* hydroxides, carbonates).

Here the incorporation of biological methods into the process (Scheme 1) may provide the key, as bioprocessing occurs at relatively low temperatures (≤30 °C), in aqueous solutions and does not involve the use of hazardous solvents or compounds such as NaOH to raise the pH due to other alkaline compounds (*e.g.* carbonates and ammonia) produced by the bacteria.^20^ In this study, bacteria were used for metal bio-recycling, as they grow quickly and are engineered more easily in comparison to other microorganisms, should the process require such optimisation. We examined the selective separation and recovery of the most relevant metals (Co, Li, Mn and Ni) present in EV LIB (NMC) leachates prepared with strong mineral acids using two bacterial species, *Shewanella oneidensis* MR-1 and *Desulfovibrio alaskensis* G20. These bacteria reportedly precipitate dissolved Mn and Ni ions in the form of Mn oxide^21^ and Ni sulfide nanoparticles (NPs)^22^ respectively and additionally, during the course of this work, we confirmed the synthesis of Co NPs by *D. alaskensis* G20. The biological mechanisms responsible for metal removal such as biosorption, bioprecipitation and bioreduction are ubiquitous but, in the main, are metal and bacterial species-specific.^23^ Metal oxido-reduction reactions resulting in nanoparticle synthesis are usually associated with the production of metal reducing and metal binding compounds such as enzymes and non-enzymatic proteins.^20^ Thus we interrogated the proteome of *D. alaskensis* G20 to gain insight into the biological pathways responsible for the precipitation of Co and Ni. It is crucial to recycle both metals as they are currently essential in the majority of EV LIB chemistries; Co is a critical element and there is an increasing demand for Ni due the rapid adoption of high-Ni cathodes.^24,25^

## Results and discussion

### Bioprecipitation of nickel, manganese, and cobalt complexes

**Fig. 1:**
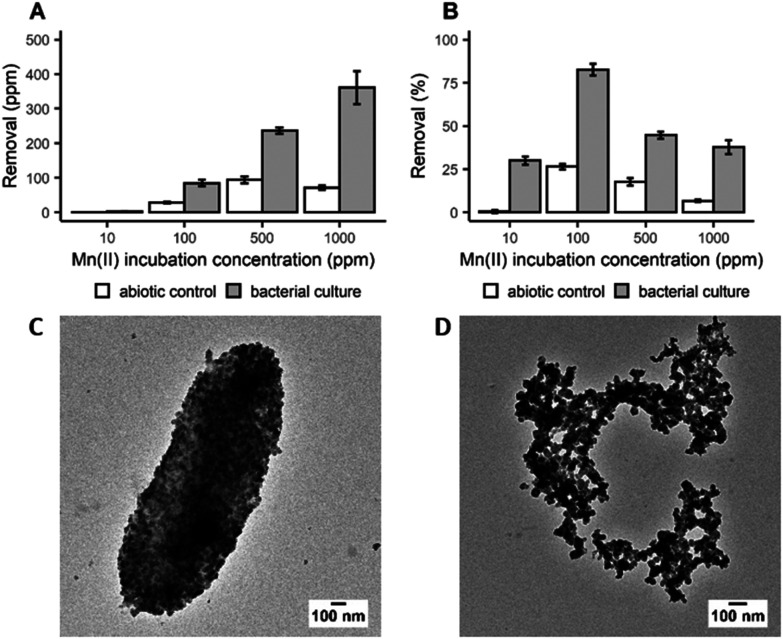
Removal of dissolved Mn^2+^ expressed as mean ± SD (*n* = 3) measured as (A) ppm and (B) percent with cell cultures of *S. oneidensis* MR-1 and abiotic control. TEM images of nanoparticles produced by *S. oneidensis* MR-1 incubated with 1000 ppm of Mn^2+^ (C) coating the bacterial cells and (D) detached from bacterial biomass.

In order to determine the efficiency of Mn bioprecipitation by *S. oneidensis* MR-1, experiments using single-metal solutions were carried out at 20 °C, over a period of 20 h. Manganese recovery was investigated in systems (pH 7–8.5) containing 10 to 1000 ppm of dissolved Mn^2+^ ions based upon the composition of real EV LIBs leachates. It was observed that while total Mn recovery increased with increasing initial Mn^2+^ concentration (Fig. 1A), the highest recovery efficiency was observed from the 100 ppm solution, with 83% of the dissolved Mn^2+^ recovered (Fig. 1B). The low recoveries at 10 ppm suggests that there is a minimum dissolved Mn^2+^ concentration threshold for recovering Mn efficiently. However, not all of this Mn recovery is associated with bacterial activity, as Mn precipitates were also detected in the abiotic control (27% recovery at 100 ppm incubation concentration). Nevertheless, taking incubation times (20 h) and cell densities into account, the removal rate obtained in this study is encouraging compared to previous work with *Shewanella putrefaciens* which required longer incubation times, up to 10 days, to achieve 80% removal at an incubation concentration of 125 ppm.^26^ Analysis by X-ray powder diffraction (XRPD) identified the white precipitate obtained at the end of the incubation period as MnCO_3_ (ESI, Fig. S1A†). Transmission electron microscopy (TEM) imaging confirmed that this biogenic MnCO_3_ was in the form of nanoparticles both coating the bacterial cells (Fig. 1C) and was also detached from cell biomass (Fig. 1D). This latter material has the greater potential for resynthesis into LIB electrode active materials.^27,28^ It is very likely that Mn^2+^ precipitated as MnCO_3_ due to carbonate species produced during bacterial growth (ESI, Fig. S2A†). The synthesis of carbonates increases the pH of the culture during bacterial growth (ESI, Fig. S2B†) supporting Mn biomineralisation and bioprecipitation. *S. oneidensis* does not produce urease, the enzyme known for mediating fungal synthesis of MnCO_3_^28^ and the molecular mechanisms responsible for the synthesis of carbonate by this bacterium are still unknown^29^ and deserve further investigation.

Following the work with Mn we extended our investigations with *S. oneidensis* MR-1 to precipitate other relevant metals, Li, Co and Ni, without success. This confirmed this bacterium was a suitable candidate for the selective recovery of Mn from LIBs leachates.

**Fig. 2:**
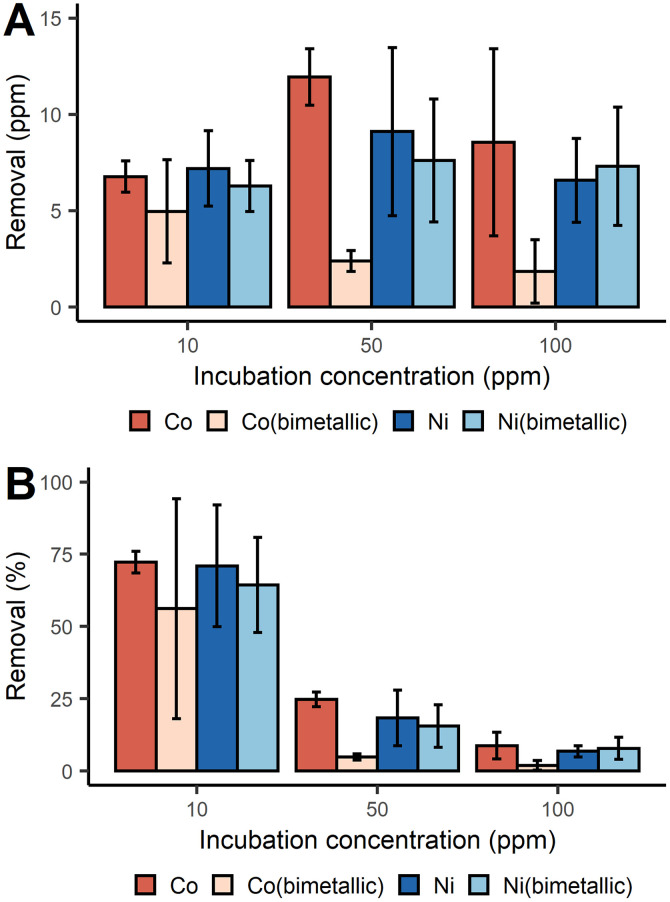
Removal of Co^2+^ and Ni^2+^ with *D. alaskensis* G20 expressed as mean ± SD (*n* = 3) measured as (A) ppm and (B) percent.

With a similar batch process approach, we demonstrated that *D. alaskensis* G20 was able to remove both Co^2+^ and Ni^2+^ from the dissolved fraction with an efficiency above 70% at 10 ppm of metal ions. The net mass recovery of Ni varied little across the different incubation concentrations (Fig. 2A). While the removal of Co did increase with higher incubation concentrations, the removal efficiency for both metals was most efficient at lower concentrations (Fig. 2B).

Unlike the Mn systems, there was no Co or Ni precipitation observed in either of the abiotic control solutions. As vehicular LIBs leachates can often contain both Co and Ni, the removal of Co and Ni from bimetallic solutions was also studied. It was found that in general the presence of Ni^2+^ significantly decreased the removal of Co^2+^ at all three incubation concentrations tested (Fig. 2A). Significant differences in the removal of Co were found between the single and bimetallic treatments (two-way ANOVA, *p*-value <0.001). The differing removal profiles of these metals underpins the existence of different biological molecules and mechanisms responsible for Co^2+^ and Ni^2+^ precipitation which were investigated with a proteomics study (ESI, proteomics analysis, Fig. S3†). For instance the abundance of the UPF0173 metal-dependent hydrolases (Dde_0151) and MJ0042 family finger-like proteins (Dde_0116) increased after 2 h incubation with 100 ppm Co^2+^ compared to the control treatment, but decreased in the bimetallic treatment. These two protein families bind Zn^2+^, but can also bind Co^2+^ without significant loss of functionality.^30,31^ To reduce the risk of mis-metallation with Ni, or perhaps to remedy such, it is understandable that a decrease in their abundance is observed and this might then also explain why Co removal from the dissolved fraction dropped when Ni^2+^ was present at concentrations ≥50 ppm.

### Characterisation of Co and Ni nanoparticles

**Fig. 3:**
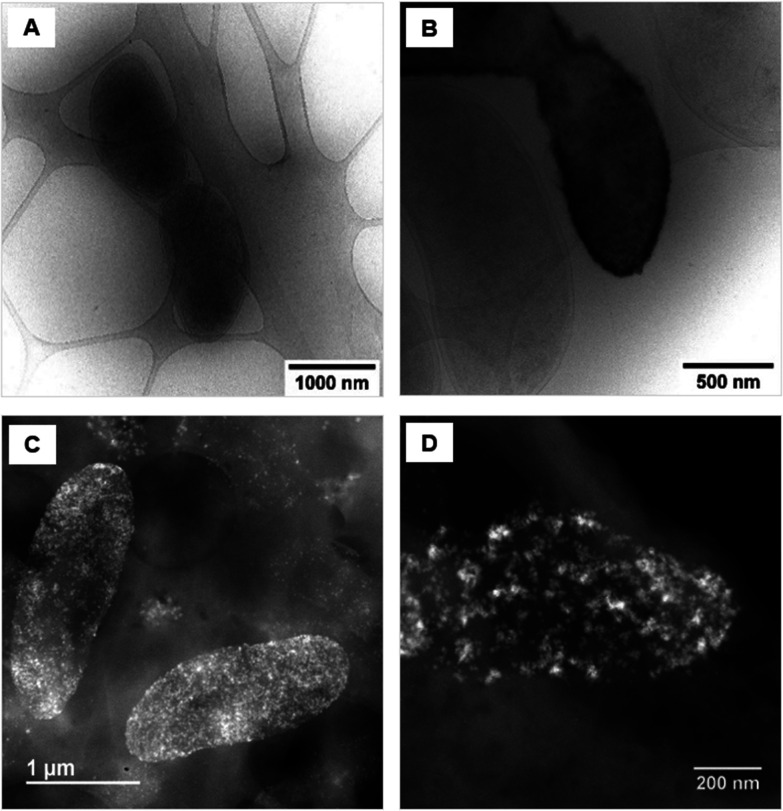
Cryo-EM images of *D. alaskensis* G20 (A) in the control treatment and (B) after incubation with 50 ppm of Co^2+^. STEM image of samples of *D. alaskensis* G20 incubated for 20 h with 10 ppm of (C) Co^2+^ and (D) Ni^2+^.

Upon examination of the bacterial cells post-treatment using cryo-electron microscopy (cryo-EM), areas of high-density were observed in the bacterial envelope of cells incubated with 50 ppm Co^2+^ (Fig. 3B) compared to cells in the control treatment (Fig. 3A).

**Fig. 4:**
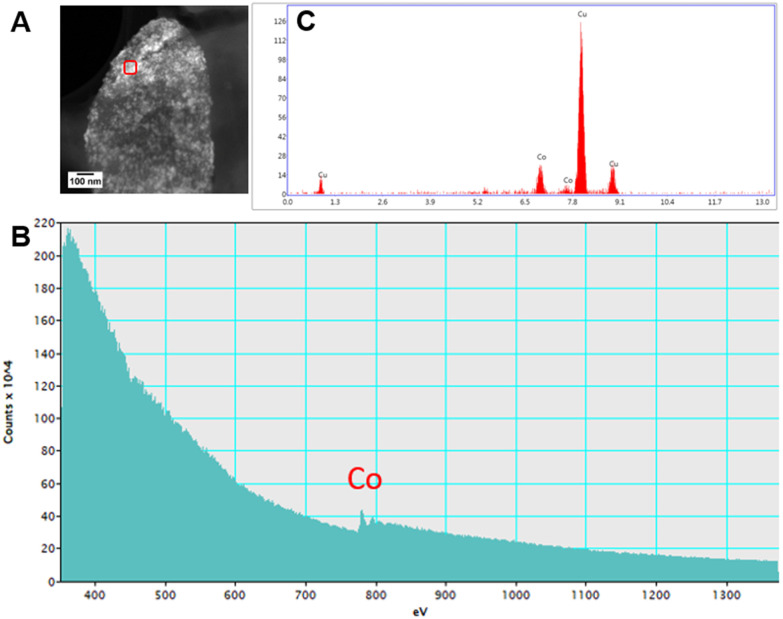
Characterisation of Co nanoparticles by STEM, EDXS and EELS. (A) STEM image, (B) EELS and (C) EDXS spectra taken from the spot highlighted with red square on image (A), on the surface of *D. alaskensis* G20 incubated for 20 h in Co^2+^ 10 ppm, and re-suspended at a final concentration of ethanol 50% v/v. Cu peaks in the EDXS are associated to the TEM grid.

**Fig. 5:**
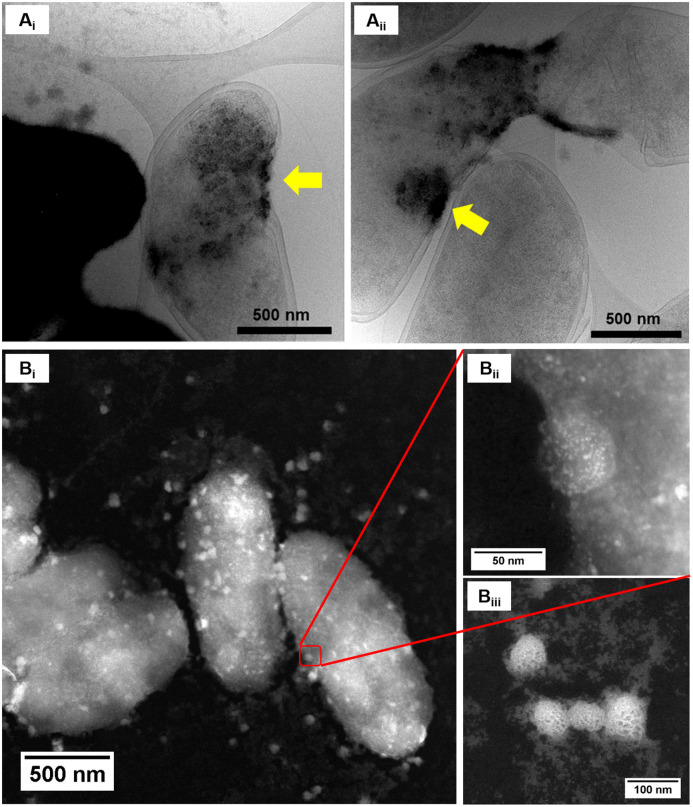
Characterisation of Co nanoparticles. (A) Cryo-EM images of *D. alaskensis* G20 incubated for 20 h in Co^2+^ 50 ppm, yellow arrows point at high-density areas in the bacterial envelope where Co-based nanoparticles were formed. (B) STEM image of mesoporous nanostructures synthesised on the bacterial envelope of *D. alaskensis* G20 cells incubated for 20 h in Co^2+^ 50 ppm.

**Fig. 6:**
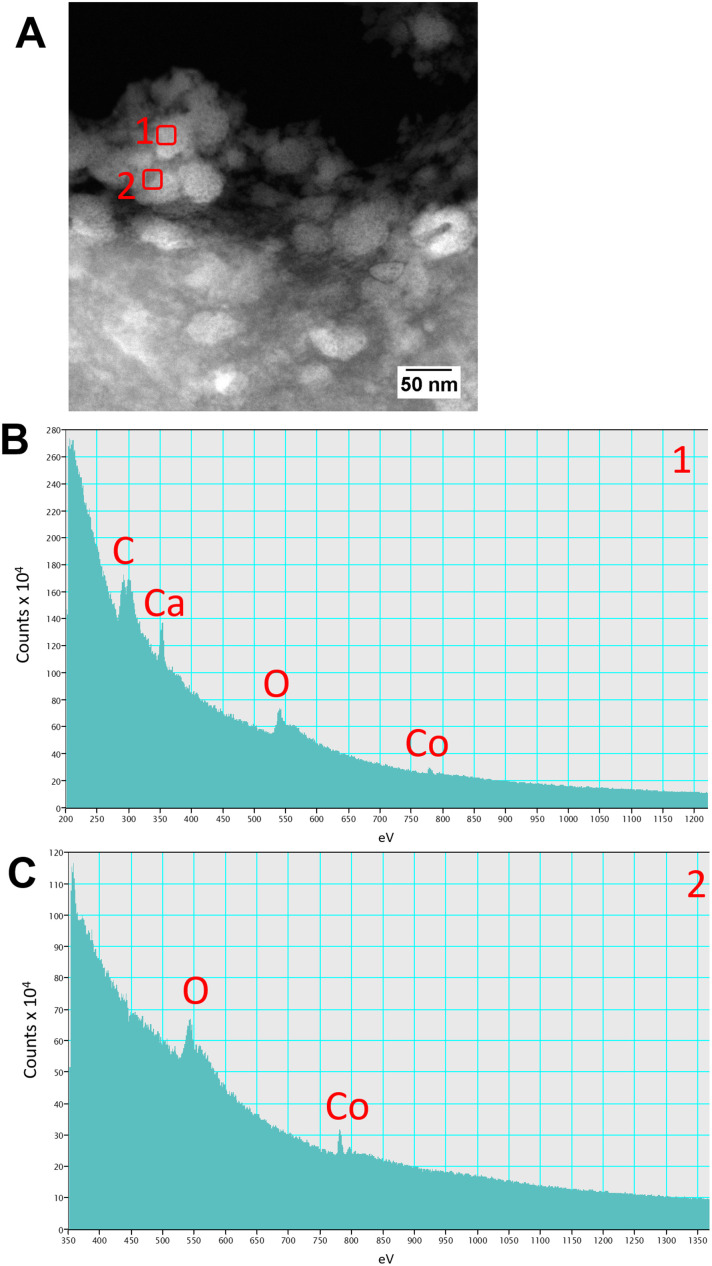
Characterisation of the mesoporous nanostructures using (A) STEM and (B and C) EELS spectra obtained from regions (A) 1 and 2 highlighted with red squares.

High resolution scanning transmission electron microscopy (STEM) imaging also showed the presence of high-density areas covering the surface of bacterial cells incubated with Co^2+^ (Fig. 3C) and Ni^2+^ (Fig. 3D) at 10 ppm, depicting the formation of metallic nanoparticles. To our delight the characterisation revealed zero-valent Co NPs attached to the cells (Fig. 4A), the first time, to the best of our knowledge, that biosynthesis of such nanoparticles of this critical metal have been reported. The absence of oxygen in the electron energy loss spectroscopy (EELS) (Fig. 4B) and sulfur in the EDXS (Fig. 4C) spectra rules out the possibility that the NPs are sulfides. The synthesis of biogenic zero-valent Co NPs by the bacterium *Geobacter sulfurreducens* had been previously speculated but not demonstrated.^32^ The biological mechanisms involved in the formation of zero-valent Co NPs are unknown and deserve further investigation that has commenced with the proteomics work presented in this study. From this we hypothesise that certain proteins, such as quinone-interacting membrane-bound oxidoreductases (ESI, Fig. S3A and B,† Dde_1113), and other redox active elements (*e.g.* cytochromes, flavins^33,34^) could be involved in the reduction of Co^2+^ to Co^0^ during the anaerobic metabolism, under negative oxidation–reduction potential conditions. Other metal-binding proteins in the membrane (ESI, Fig. S3,† Dde_0155, Dde_2208, Dde_3518) may play a role in the nucleation of Co resulting in nanoparticle formation and stabilisation. Cryo-EM images show that Co nanoparticles are synthesised in the bacterial envelope eventually compromising the integrity of the membrane (Fig. 5A). The bacterial envelope under the STEM presented different degrees of degradation depending on the fixative used, ethanol 50% v/v was less aggressive than acetone 50% v/v. The images obtained from samples of *D. alaskensis* G20 incubated with Co salts and fixed with acetone showed mesoporous nanostructures of a diameter ∼50 nm (Fig. 5B and 6A). EELS analysis confirmed the presence of Co in these mesoporous nanoparticles. The increased concentration of metal-binding proteins during incubation with Co in different locations of the bacterial envelope (ESI, Fig. S3†) such as Dde_2670 in the inner membrane and Dde_0155, Dde_2208, Dde_1113, and Dde_3518 in the periplasm, support the hypothesis that these Co-based nanostructures are formed due to biological processes. We could not confirm the oxidation state of Co in this instance because elemental edges associated to C, Ca and O were also present in the EELS spectra (Fig. 6B and C). The presence of Ca could be attributed to accumulation as a result of the direct electron transfer from cytochromes and hydrogenases^35^ or alternatively may be associated to the degraded bacterial envelope surrounding the NPs.^36^ In addition to the biological mechanisms of metal bioprecipitation, the formation of nanocrystalline cobalt and nickel sulfides must be considered due to the presence of biogenic hydrogen sulfide (H_2_S).^37^ Whilst the cells were thoroughly washed and resuspended in nutrient-free buffer prior to the beginning of the metal ion removal experiments, biogenic H_2_S was produced ([H_2_S] <160 μmol L^−1^) (ESI, Fig. S4†) during maintenance of the bacterial cell steady-state.^38^ The formation of nanoparticles made of Co and S (ESI, Fig. S5A†), and Ni and S (ESI, Fig. S5B†) on the surface of the bacterial envelope was confirmed by STEM/EDXS. Precipitation of Co and Ni as nanocrystalline metal sulfides by the activity of sulfate reducing bacteria (SRB) has been reported previously and is attributed to the presence of biogenic H_2_S.^37^ However, the presence of sulfur in the nanoparticles could also be due to the presence of cysteinyl ligands in relevant proteins binding iron-sulfur clusters. Some proteins binding 4Fe–4S were significantly more abundant after incubation with Ni^2+^ (ESI, Fig. S3B and C,† Dde_0718, Dde_2176, Dde_1830, Dde_2943). There is a possibility that the iron sites within these metalloproteins have been replaced by Ni^2+^ and/or Co^2+^, as observed in rubredoxins-related studies,^39^ and thus end up serving as an anchor and metal nucleation site for the nanoparticles synthesised as observed on the bacterial outer membrane.^40^ Metal removal by H_2_S is relatively efficient and in the bacterial cell-free supernatant was above 70% due to the presence of dissolved H_2_S (24.5 mmol L^−1^), however it exhibits no selectivity for Co or Ni (ESI, Fig. S6†).^41^

### Selective bioprecipitation of dissolved Mn from vehicular LIBs leachates

To investigate metal removal/precipitation from vehicular battery leachates, two commercial LIB cathodes (Nissan Leaf) were leached with two different mineral acids, H_2_SO_4_ or HCl, both widely used in hydrometallurgy.^42^ These cathode materials contained either Li, Ni, Mn and Co in a ratio of 50% nickel, 30% manganese, and 20% cobalt (NMC-532), or a mixture of approximately 70% lithium manganese oxide spinel (LMO) with 25% lithium nickel manganese cobalt oxide,^43^ referred to hereafter as A1C and B1C, respectively. The concentration of metals (Al, Co, Mn, Li and Ni) in these LIB leachates was dependent upon the origin of the cathode material, solvent used and leaching temperature (ESI, Table S1†). Prolonged leaching times, from 5 to 30 minutes in 0.1 M H_2_SO_4_ at 50 °C doubled the concentration of Co, Li and Mn, and increased Al and Ni concentrations even further. The final dissolved metal composition of the leachates was very different according to the battery type, cathode chemistry and acid used. The leaching data for these cathodes in H_2_SO_4_ and HCl at 20 °C is available in ESI, Fig. S7.†

**Fig. 7:**
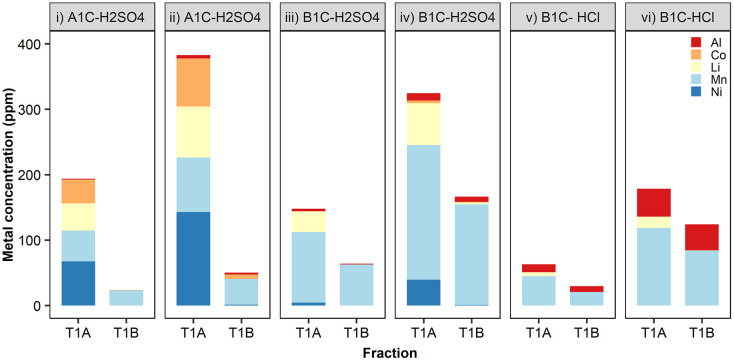
Bioprecipitation of metals contained in vehicular LIBs using *S. oneidensis* MR-1. Selected leachates: cathode A1C at 50 °C in 0.1 M H_2_SO_4_ for (i) 5 min and (ii) 30 min; cathode B1C at 50 °C in 0.1 M H_2_SO_4_ for (iii) 5 min and (iv) 30 min; cathode B1C at 20 °C in 0.1 M HCl for (v) 5 min and (vi) 300 min. Metal concentration in the raw leachate (T1A) and metal removal/precipitated (T1B) expressed as the mean (*n* = 3 biological replicates).

The specificity for dissolved Mn and the removal rates achieved by *S. oneidensis* MR-1 with vehicular leachates were in agreement with the results we obtained in the previous experiments using metal salts, *i.e.* higher concentrations of metal ions resulted in greater total metal precipitates. The capability exhibited by this bacterium for the selective precipitation of Mn^2+^ out of the mixed metals contained in the crude leachates is extremely relevant for establishing the principles of a bio-separation process (Fig. 7, ESI Table S2†). The removal efficiency of Mn peaked at 75% (154 ppm) from leachates of the cathode material B1C dissolved in 0.1 M H_2_SO_4_ for 30 minutes at 50 °C (Fig. 7iv). The removal of the other metals was low, generally well below 5% total precipitated mass, except for Co when present at concentrations above 70 ppm (Fig. 7ii) and Al contained in HCl leachates (Fig. 7v and vi). Fortunately, a recent advance using selective hydrometallurgical methods combined with high-intensity ultrasonication can now be employed to provide leachates with a lower concentration of Al through delamination of the active materials from the foil current collectors.^10^

### Two bacterial treatment bioprocessing approach

**Scheme 2:**
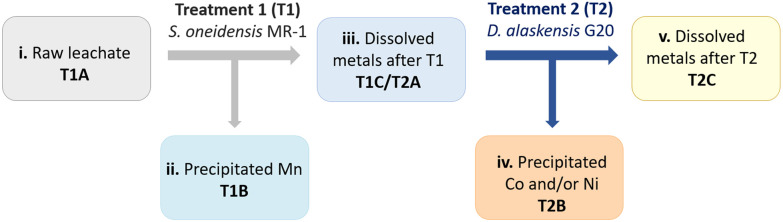
Two-bacterial metal bio-separation and recycling approach. **T1A**: initial dissolved metals from the raw leachate; **T1B**: precipitated metals after treatment 1(T1); **T1C**: metals remaining in dissolved fraction after T1; **T2A**: initial concentration of dissolved metals (different volumes of T1C) before treatment 2 (T2); **T2B**: precipitated metals after T2; **T2C**: metals remaining in the dissolved fraction after T2.

In order to ensure a complete and selective bio-recycling process, a two-bacterial bioprocessing approach was developed by using *S. oneidensis* MR-1 to selectively precipitate out Mn (treatment 1, Scheme 2), followed by treatment of the stripped solution using *D. alaskensis* G20 to precipitate Ni and/or Co (treatment 2, Scheme 2).

**Fig. 8:**
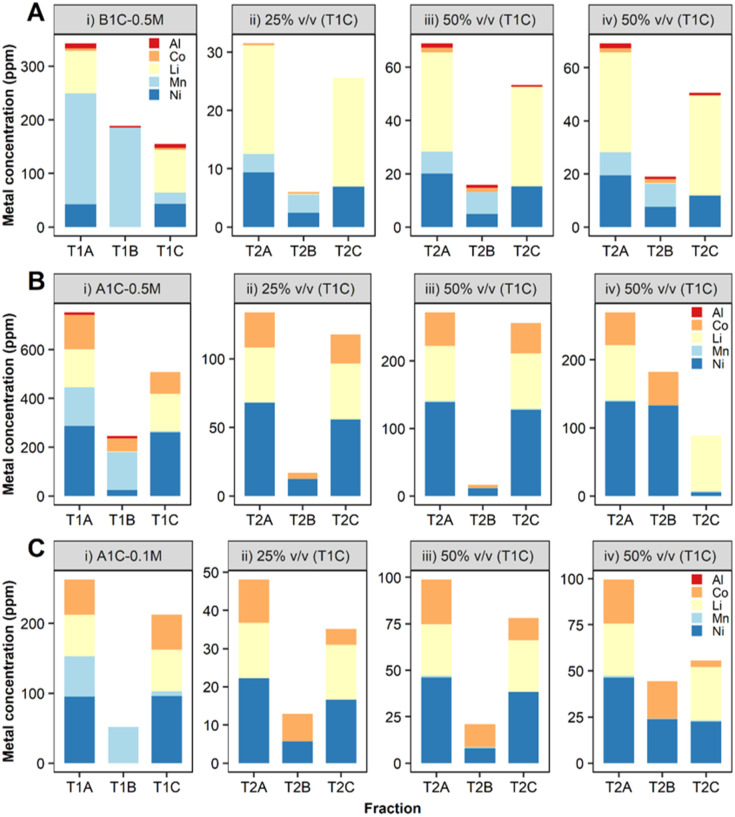
Bio-separation of metals contained in leachates prepared with (A) B1C 0.5 M H_2_SO_4_ (B) A1C 0.5 M (C) A1C 0.1 M H_2_SO_4_. Metal concentration expressed as the mean (*n* = 3 biological replicates) associated to different stages of (i) T1 and T2 (ii and iii) cell-only treatment (thoroughly washed bacterial cells in MOPS buffer) and (iv) whole bacterial cell culture.

To enable the use of leachates with higher acid content (0.5 M H_2_SO_4_), a carbonate–bicarbonate buffer was added to *S. oneidensis* MR-1 cell culture. Small-scale (2 ml) experiments confirmed this buffer would aid the biological removal of Mn from leachates prepared in 0.5 M H_2_SO_4_ (ESI, Fig. S8†). The results obtained were very encouraging with the B1C leachate (Fig. 8A(i)-T1B, ESI Table S3†) as the precipitation of Mn was enhanced to 89% and the dissolved Mn present in the downstream fraction (T1C) was reduced to just 10 ppm (mass balance ESI Fig. S9†). A higher (97%) Mn removal was achieved from the A1C leachate, however the precipitated Mn exhibited lower purity (63% total precipitated metal mass) due to co-precipitation of Al (4%), Co (22%) and Ni (10%) (Fig. 8B(i)-T1B, ESI Table S4,† mass balance Fig. S10†).

#### Recovery of Co and Ni

After precipitation of Mn from the battery cathode leachates using *S. oneidensis* MR-1, the fractions containing the remaining dissolved metals (T1C) for both B1C and A1C leachates were processed with *D. alaskensis* G20 to precipitate Ni and Co. The initial concentration of dissolved Co and Ni (fraction T2A) was lower in the B1C leachate (Fig. 8A, ESI Table S3†) than in the A1C (Fig. 8B, ESI Table S4†) due to the differences between the cathode chemistries. When working with the whole bacterial culture, Co and Ni extraction was improved compared to the cell-only treatment (thoroughly washed bacterial cells in MOPS buffer). This could be related to higher concentrations of H_2_S enhancing metal precipitation as Ni and Co sulfides. While this increase in precipitation is a positive result for metal recovery, it does however introduce impurities into the process *via* the presence of multiple metal species that must be separated afterwards. Therefore, optimization of the cell-only treatment was investigated further to demonstrate that by adjusting the concentrations and ratios between Co and Ni, the metal specificity of *D. alaskensis* G20 can be altered. Higher selectivity towards Ni removal was observed when this metal was present at concentrations above 50 ppm (conditions with A1C 0.5 M H_2_SO_4_ leachate, Ni and Co were in the ratio 8 : 3) in agreement with the results obtained during the bimetallic experiments at 50–100 ppm. The release of extracellular proteins with high Ni-affinity as a mechanism of bacterial stress response caused by this metal may also explain differences between Co and Ni removals.^44^ Our proteomics study showed proteins that might be involved in metal reduction processes, such as oxidoreductases,^45,46^ which were significantly more abundant after 20 h incubation with 10 ppm of Ni^2+^ compared to the treatment with Co^2+^ (ESI, Fig. S3C†) confirming a distinctive cellular response depending on the metal. Some of these oxidoreductases, such as the FAD/NAD (P)-binding domain protein (Dde_1381) and the FAD-dependent pyridine nucleotide-disulfide oxidoreductase (Dde_2176), are classified within the xenobiotics biodegradation and metabolism pathways and might be responsible for reducing Ni^2+^ into a less toxic form. Since Ni was present at toxic concentrations for *D. alaskensis* G20 in the A1C 0.5 M H_2_SO_4_ leachate, the removal of Ni and Co from a leachate (A1C, 0.1 M H_2_SO_4_) containing lower concentrations of both metals was investigated (Ni : Co ratio of 2 : 1). This time *D. alaskensis* G20 showed preference for precipitating Co, and by increasing the volume of leachate from 25 to 50% v/v, the removal increased by 70% for Co, 12.2 ppm, and 40% for Ni, 7.9 ppm (Fig. 8C, ESI Table S5†). The removal yields of Co and Ni presented here are still low as experiments have been developed in a single batch process in 2 ml volumes as a first step to show the fundamentals of the approach and to provide benchmark data for process optimisation. The Li that remains in the dissolved fraction at pH > 8, with similarities in terms of composition and concentration to some lithium salar brines,^47^ could then be recovered by chemical precipitation as Li_2_CO_3_.

#### Scale-up

**Fig. 9:**
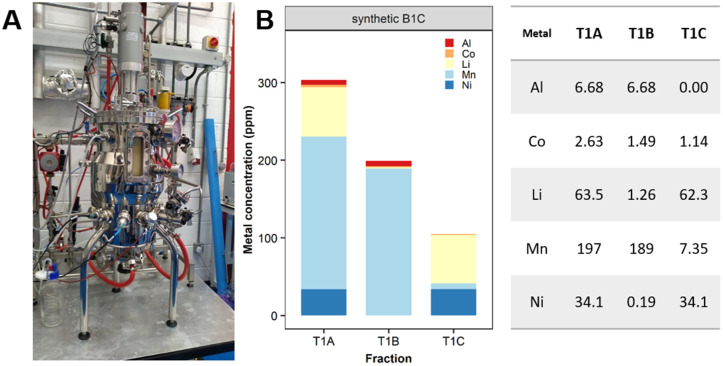
Scale-up of Mn removal with synthetic leachates. (A) Semi-pilot reactor vessel (FlexBio, IBioIC) and (B) Mn recovery (ppm) from a synthetic leachate based on B1C cathode dissolved in 0.5 M H_2_SO_4_.

The feasibility of scaling-up the first treatment (T1) of the bioprocess was successfully confirmed with a semi-pilot bioreactor (30 L vessel) (Fig. 9) using model metal solutions comparable to the metal concentration and acid composition of the leachate obtained from dissolving the B1C in 0.5 M H_2_SO_4_. The removal of Mn was 96.3% demonstrating the scalability of the process. Precipitated metals and biomass were separated from the dissolved fraction by centrifugation. The sedimentation rate of Mn, in the form of bio-MnCO_3_, was faster than bacterial mass due to its higher density. Thus two distinct layers were formed (ESI, Fig. S11†).

## Conclusion

The need for more efficient and greener methods for recycling metals contained in LIBs is driving research to consider the application of less conventional methodologies. For decades microorganisms have been used extensively in the areas of metal bioremediation and bioleaching however their uses for metal bio-separation are still in the early stages. Our work with two different bacterial strains shows the potential for the separation and recovery of all the relevant metals contained in LIBs. First *S. oneidensis* MR-1 precipitates dissolved Mn followed by the use of *D. alaskensis* G20 for the recovery of Co and/or Ni, leaving Li in the downstream leachate as could be found in a lithium salt brine. The results presented here show the principles for establishing a bio-based technology and will be used as the benchmark to define areas of research needed for enhancing removal yields and improving metal selectivity. To date there is a wide range of synthetic biology tools available for manipulation of *S. oneidensis* MR-1 that could be applied for improving the removal efficiency of Mn^2+^. The removal of Co and Ni was higher with whole bacterial culture treatment however no selectivity for either metal was observed. In contrast, the utilisation of bacterial cell-only treatment offers greater advantages such as better control of the physicochemical properties of the nanoparticles produced and the potential for enhancing the specificity for Co or Ni by using *D. alaskensis* G20 engineered strains with their design informed by the proteomics analysis provided herein. Biological approaches are ideally suited for implementation alongside existing technologies and could form part of battery recycling processes to provide a sustainable incentive for industry and as a solution to fit EV LIB technology within a circular economy. The implementation of life cycle assessments (LCAs) will ultimately be the tool to compare the sustainability of the methodologies for recycling end of life LIBs.^48^

Another exciting finding achieved in this work was the synthesis of novel nanoparticles. The identification of biogenic zero-valent Co NPs shows the potential of biology for producing unique nanoparticles with perhaps novel physicochemical properties. This finding provides us with new insight into the nanoparticle synthesis pathways of *D. alaskensis* G20 and deserves further investigation due to the relevance of bio-based Ni and Co nanoparticles as electrocatalysts in Hydrogen Evolution Reactions (HER).^49,50^

## Experimental

### Bacterial cultures preparation

*S. oneidensis* MR-1 was cultured aerobically in Luria Bertani (LB) media without NaCl, LB no salts (LBNS).^51^ Overnight cultures (5 ml) were used to inoculate larger volumes (100–200 ml) at 200 rpm and 20 °C until stationary phase (OD_600_ ∼ 6). Cultures of *D. alaskensis* G20 were grown in Postgate Media C (PGMC) as described elsewhere,^22^ washed with MOPS buffer (pH 7.5, 10 mM) and re-suspended in fresh MOPS buffer (OD_600_ = 1).

### Measurement of dissolved carbonate

Bacterial cultures of *S. oneidensis* MR-1 were collected at different growth levels (monitored by OD_600_) and centrifuged for 20 min at 4500 rpm, 20 °C. Afterwards supernantants were filter-sterilised (∅ < 0.2 μm) and stored at 4 °C until analysis. The carbonate analysis of the bacterial supernatant was performed by coulometric titation using a CM 5012 CO_2_ coulometer (UIC Inc., Joliet, IL, USA) and CM 5130 acidification unit. The coulometer measured the inorganic carbon mass (*M*_IC_) which was converted to the equivalent mass of CO_3_^2−^ (1 μg C = 5 μg of CO_3_^2−^, under the assumption that most of the inorganic carbon is in a bicarbonate/carbonate form) using eqn (1) where *V* (ml) is the volume of the sample:

1 [CO_3_^2−^] = (*M*_IC_ × 5)/*V*

### Measurement of dissolved H_2_S

The concentration of H_2_S was measured in fully grown cultures of *D. alaskensis* G20 and cells resuspended in MOPS in the absence of metal using a H_2_S microsensor following manufacturer's guidelines (UNISENSE).

### Bioprecipitation of nickel, manganese, and cobalt complexes

Stock metal solutions were made in ultrapure water to a concentration of 10 g L^−1^ using Co·Cl_2_·6H_2_O, LiCl, MnSO_4_·H_2_O and NiCl_2_·6H_2_O as a source of Co^2+^, Li^+^, Mn^2+^ and Ni^2+^ ions respectively. Bacterial cell cultures and abiotic controls, fresh media and buffers, were incubated with metal solutions in 15 ml falcon tubes at a final volume of 2 ml, pH was not adjusted. *S. oneidensis* MR-1 treatments took place aerobically (20 °C and 200 rpm), whereas *D. alaskensis* G20 treatments took place in an anaerobic atmosphere (10 % CO_2_, 10 % H_2_ in N_2_ atmosphere, static, 30 °C).

#### Metal removal analysis

After a 20 h incubation 1 ml aliquot samples were collected for metal removal analysis. A volume of 100 μl of this aliquot was acidified with 900 μl of 20% HNO_3_ (v/v) to analyse for the total metal concentration. The remaining sample was centrifuged (2 h, 20 000*g* and 4 °C), and 100 μl of supernatant (dissolved fraction) acidified with 900 μl of 20% HNO_3_ (v/v). The acidified samples were digested for 5 h at 80 °C and diluted in ultrapure water prior to inductively coupled plasma optical emission spectrometry (ICP-OES) analysis on an Optima 8300 (PerkinElmer).^51^ Metal removal (%) from the dissolved fraction was calculated as the difference between the total concentration (*T*_c_) of the metal added and the concentration of metal that remained in the dissolved fraction (*D*_c_) as depicted in eqn (2):

2 Metal removal (%) = ((*T*_c_ − *D*_c_)/*T*_c_) × 100

Differences between Co and Ni precipitation across different conditions were analysed with ANOVA.

### Selective bioprecipitation of dissolved Mn from vehicular LIBs leachates

The LIB leachates used were prepared with two different cathode materials: A1C (NMC-532) and B1C (70% lithium manganese oxide spinel (LMO) with 25% lithium nickel manganese cobalt oxide) obtained from uncycled electric vehicle batteries. The cathode materials were dissolved in H_2_SO_4_ (0.1 M) at 50 °C over a period of 5 to 30 minutes with no agitation. Leachates of B1C cathode were also produced in HCl (0.1 M) at 20 °C for up to 5 h with no agitation. The bioprecipitation of metals contained in vehicular LIBs leachates was investigated following the same methodology used to investigate metal removal from single-metal solutions. Cultures of *S. oneidensis* MR-1 were incubated with 10% v/v of raw leachates (2 ml final volume) prepared in H_2_SO_4_ (0.1 M) and HCl (0.1M) for 20 h at 20 °C and 200 rpm. The concentration of metals was analysed by ICP-OES.

### Nanoparticle characterisation

#### X-Ray powder diffraction

Samples of *S. oneidensis* MR-1 incubated with Mn^2+^ were collected by centrifugation at 20 °C and 4500 rpm for 10 min. Pellets containing bacterial biomass and bioprecipitated Mn were washed consecutively with ultrapure water, 70% v/v ethanol and then ultrapure water to remove any remaining dissolved forms of Mn and to inactivate the bacterial cells. Washed pellets were then freeze-dried, ground in a pestle and mortar and mounted in a silicon deepwell mount prior to XRPD analysis. XRPD data was collected using a Bruker D2 Phaser X-ray powder diffractometer, configured in reflection geometry, using Cu Kα radiation (1.541 Å) and a LynxEYE X-ray detector. Data was collected over the two theta range 5–60° for 15 minutes.

#### STEM, EDXS and EELS

Aliquots of bacterial cultures were collected after 20 h incubation with metal salts, and resuspended with ethanol or acetone (both 50% v/v) to inactivate bacterial processes. Afterwards samples were stored and preserved at 4 °C in an anaerobic atmosphere until characterisation. Nanoparticle production was investigated by using high-resolution aberration-corrected STEM, to resolve their density and shape, and EDXS and EELS were used to investigate their elemental distributions. Samples for TEM were dispersed for 10 minutes in an ultrasonic water bath and then small drops of nanoparticle solution were taken on to the carbon coated copper grid. Ni and Co nanoparticle characterisation was carried out on these thin films using the aberration-corrected JEOL JEM-2100F at 200 kV. ADF-STEM images were obtained using a JEOL annular field detector with a fine-imaging probe and a current of 50 pA with a convergence semiangle of ∼25 mrad and an ADF detector inner angle of 50 mrad. EELS was recorded using a Gatan GIF Quantum SE (model 963). Measurements were performed at a total energy resolution of ∼3 eV, determined by measuring the full width at half-maximum (fwhm) of the zero-loss peak. The following conditions were chosen for the EELS spectra acquisition: convergence semi-angle 30 mrad, collection semi-angle 100 mrad, exposure time 0.05 s, dispersions of 0.5 and 1 eV per ch, and probe size <0.5 nm. EDXS was recorded using EDAX Octane T Optima system, with a windowless 60 mm^2^ SDD EDX detector.

#### Cryo-EM

Cryosamples were prepared immediately after incubation with and without metal salts using a Vitrobot Mark IV (Thermo Fisher Scientific). Teflon sheets were used between the blotting pads and blotting paper to reduce contamination of the pads. 4 μl of sample was applied to freshly glow discharged lacey carbon grids (300 mesh copper grids, C269/C TAAB), blotted and plunged automatically into liquid ethane. Freezing conditions were set to: blotting force-1, blotting time 2 s, drain time 1 s and 5 s wait time with conditions in the sample preparation chamber set to 100% humidity and room temperature. Samples were stored under liquid nitrogen until imaging. Cryo-EM was performed using a Tecnai F20 200 kV electron microscope (Thermo Fisher Scientific). Samples were loaded into the EM using a cryoholder model 626 (Gatan). Imaging was carried out under low dose conditions, with a defocus value of −5 μm. Images were acquired using a CMOS F816 camera (TVIPS), using 8 kb2 settings.

### Two bacterial-treatment bioprocessing approach

The different stages of the bioprocess approach are summarised graphically in Scheme 2 showing the two-bacterial treatments and the resulting fractions involved. The first treatment (T1) consisted of processing the raw leachate mixed with *S. oneidensis* MR-1 to precipitate mainly Mn (T1B). Afterwards the fraction containing the remaining dissolved metals (T1C) was further processed with *D. alaskensis* G20 (treatment 2, T2) to precipitate Ni and/or Co. The study was developed with leachates prepared with A1C and B1C cathode material delaminated with H_2_SO_4_ (0.5 M, 20 min at 50 °C). Carbonate–bicarbonate buffer (final concentration 91 mM) was added to the *S. oneidensis* MR-1 cell suspension just before the addition of the acidic leachate (10% v/v). Once treated with *S. oneidensis* MR-1 the precipitated metals (T1B) were separated from the metals in the dissolved fraction (T1C) by centrifugation (10 min at 4500 rpm). Fraction T1C was filter-sterilised (∅ < 0.2 μm) before incubation with *D. alaskensis* G20 cells (cell-only treatment, OD_600_ = 2, resuspended in MOPS buffer, pH 7.5) for 20 h at different concentrations of the pretreated leachate (T1C), by volume, 25% and 50%, labelled as T2A. The metal removal using the whole bacterial culture of *D. alaskensis* G20 (cells and extracellular matrix) was investigated with 50% v/v of leachate. Removal of dissolved metal was calculated as the difference between the total concentration of metal and the remaining concentration in the supernatant after centrifugation as described previously (eqn (2)) using ICP-OES.

#### Scale-up

The scale-up of the first step of the bioprocess was investigated with a semi-pilot 30 L bioreactor vessel (Industrial Biotechnology Centre (IBioIC) FlexBio) and model metal solutions prepared in 0.5 M H_2_SO_4_ mimicking the metal composition of the leachate obtained from dissolving the B1C in 0.5 M H_2_SO_4_. Once an 18 L culture of *S. oneidensis* MR-1 (final OD_600_ = 5) was grown in LBNS the carbonate–bicarbonate buffer (final concentration 91 mM) was added to the culture followed by the addition of the acidic metal solution (2 L). After 20 h incubation, aliquots were collected for metal removal and selectivity analysis.

## Author contributions

L. E. H. conceived and directed the project, V. E. B. planned and conducted experimental work, with support from G. M. H. A. and N. D. B. were responsible for STEM, EDXS and EELS analysis. Automotive LIB leachates were supplied by J. H. X-ray powder diffraction analysis was undertaken by C. K. and CryoEM by M. W. T. All authors contributed to discussions and manuscript writing.

## Conflicts of interest

There are no conflicts to declare.

## Supplementary Material

GC-024-D2GC02450K-s001

GC-024-D2GC02450K-s002
