# Supplementary material for: Selective bacterial separation of critical metals: towards a sustainable method for recycling lithium ion batteries
Source: Green Chem. 2022 Oct 17;24(21):8512–22. doi: 10.1039/d2gc02450k (PMC9621301; doi:10.1039/d2gc02450k)
Supplement: GC-024-D2GC02450K-s001 [file GC-024-D2GC02450K-s001.pdf]

Electronic Supplementary Material (ESI) for Green Chemistry.
This journal is © The Royal Society of Chemistry 2022

Supplementary Information

Selective bacterial separation of critical metals: towards a sustainable method for recycling lithium ion batteries

Virginia Echavarri-Bravo[a,e], Houari Amari[‡b,e], Jennifer Hartley[c,e], Giovanni Maddalena[a,e], Caroline Kirk[d], Maarten W. Tuijtel[§a], Nigel. D. Browning[b,e,f,g], Louise E. Horsfall[*a,e]

[a]School of Biological Sciences, University of Edinburgh, Edinburgh, EH9 3FF, UK.

[b]Department of Mechanical, Materials and Aerospace Engineering, University of Liverpool, Liverpool, L69 3GQ, UK.

[c]School of Chemistry, University of Leicester, Leicester, LE1 7RH, UK.

[d]School of Chemistry, University of Edinburgh, Edinburgh, EH9 3FJ, UK

[e]Faraday Institution (ReLiB project), Quad One, Harwell Science and Innovation Campus, Didcot, UK

[f]Sivananthan Laboratories, 590 Territorial Drive, Bolingbrook, IL 60440, USA

[g]Physical and Computational Sciences Directorate, Pacific Northwest National Laboratory, P.O. Box 999, Richland, WA 99352, USA

[‡]Present address: Leibniz Institute for Crystal Growth, Max-Born-Str. 2, 12489 Berlin, Germany.

[§]Present address: Department of Structural Biology, Max Planck Institute of Biophysics, Max-von-Laue Strasse 3, 60438 Frankfurt am Main, Germany;

*Corresponding author. Email: Louise.Horsfall@ed.ac.uk

Contents of this document

Fig. S1. 2

Fig. S2 2

Proteomic analysis of the bacterial response to Ni and Co 1

Fig. S3. 2

Fig. S4 3

Fig. S5 3

Fig. S6. 4

Fig. S7. 4

Fig. S8. 5

Table S1. 6

Table S2. 7

Table S3. 8

Fig.S9 8

Table S4. 9

Fig.S10 9

Table S5. 10

Fig. S11. 11

References 11

Other Supplementary Data for this manuscript include the following:
Proteomics data can be accessed at https://doi.org/10.7488/ds/3130.

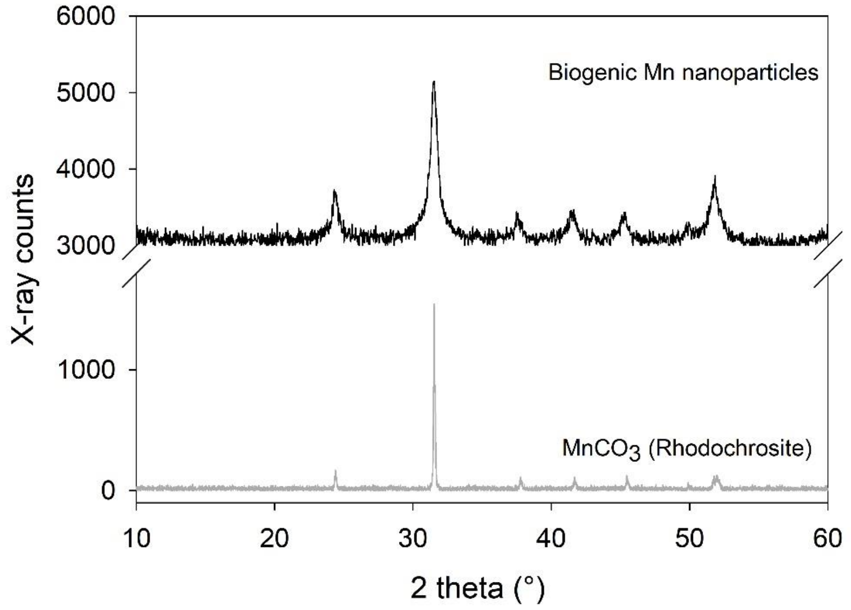

Fig. S1 XRPD data comparing the bioprecipitated Mn as $MnCO_3$ with a standard pattern of $MnCO_3$.

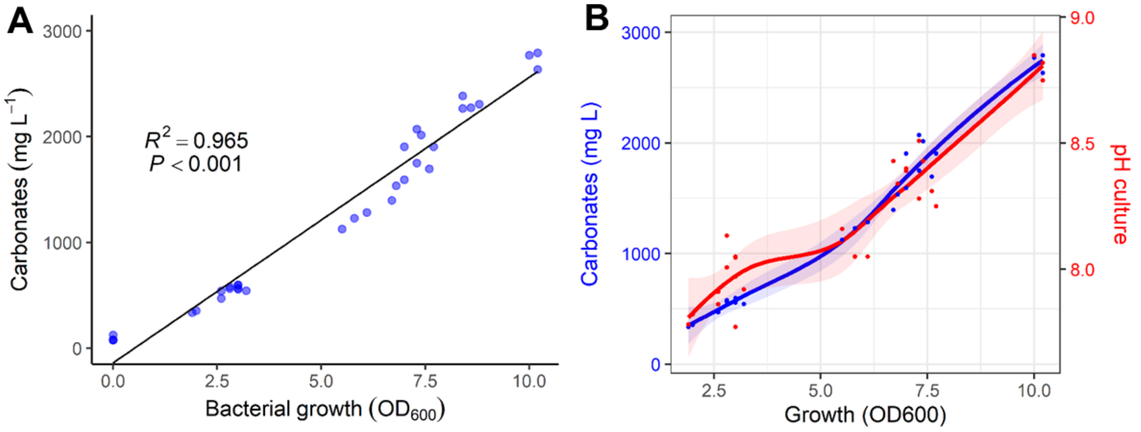

Fig. S2 (**A**) Linear correlation between bacterial growth measured as OD_{600} and carbonate production and (**B**) evolution of carbonate concentration and pH with bacterial growth (OD600).

Proteomic analysis of the bacterial response to Ni and Co

Methodology

Proteomics analysis was designed based on the removal rates of Co and Ni and considered the following factors: 1) three different metal combinations, single metal treatments (only Co^{2+} or Ni^{2+}) and bimetallic (Co^{2+} and Ni^{2+} at equal concentrations) to mimic the removal experiments; 2) two different metal concentration levels (10 and 100 ppm) and 3) two different time points (2 and 20 h) to investigate the dynamics of proteins production.

D. alaskensis G20 cultures for proteomic analysis were grown and washed in MOPS buffer similarly to the metal removal studies and previous proteomics analysis with this organism.[1] After 2 and 20 h incubation cell cultures were collected by centrifugation, the supernatants discarded and cell pellets stored at -75 °C until proteomic analysis.

ESI-HPLC-MS/MS analysis Samples were reconstituted in 8 M urea and protein concentration was determined by Bradford protein assay (Biorad). 30µg of samples were digested using S-Trap™ (ProtifFi) following manufacturers protocol. After speed-vacuum drying, peptide samples were re-suspended in MS-loading buffer (0.05% v/v trifluoroacetic acid in water) to 1µg $µl^{-1}$ final concentration and then filtered using Millex filter before HPLC-MS analysis. 5µl of a 1 to 1 dilution (in 0.05% TFA) was injected for analysis. Nano-ESI-HPLC-MS/MS analysis was performed using an online system, the nano-HPLC (Dionex Ultimate 3000 RSLC, Thermo-Fisher) coupled to a QExactive mass spectrometer (Thermo-Fisher) with a 300µm x 5 mm pre-column (Acclaim Pepmap, 5µm particle size) joined with a 75 µm x 50 cm column (EASY-Spray, 3 µm particle size). The nano-pump was run using solvent A (2% Acetonitrile in water 0.1% formic acid) and solvent B (80% acetonitrile, 20% water and 0.1% formic acid) and peptides were separated using a multi-step gradient of 2–98% buffer B at a flow rate of 300 nl min^{-1} over 90 minutes.

Data process and analysis Progenesis (version 4 Nonlinear Dynamics, UK) was used for LC-MS label-free quantitation and data normalisation and analysis. Filtering was carried out so that only MS/MS peaks with positive charges of 2, 3 or 4 were taken into account for the total number of 'features' (signal at one particular retention time and m/z) and only the five most intense spectra per 'feature' were included. MS/MS spectra was searched using MASCOT Version 2.4 (Matrix Science Ltd, UK) against a custom *D. alaskensis* G20 database with maximum missed-cut value set to 2 as in previous proteomics work with this organism.[1] For convention the protein identifier (protein ID) used in the present work has been given the name of its encoding gene which starts with letters "Dde_" followed by four numeric digits. The updates related to genes and protein annotations can be found at the KEGG database (https://www.genome.jp/kegg/). From the Progenesis exported results sheet, differentially expressed proteins were considered significant if the p-value was less than 0.05 (ANOVA) and if the number of peptides used in quantitation per protein was equal to or more than 2. Heat maps were created using R (https://cran.r-project.org/) after log10 transformation of the normalised abundance data sets.

Proteomics results

To fully develop our understanding of a biological separation process we investigated the biological molecules and mechanisms responsible for Co and Ni nanoparticle synthesis. The proteins produced by *D. alaskensis* G20 after incubation with Co^{2+} and/or Ni^{2+} were analysed by Electrospray Ionisation Mass Spectrometry (ESI)-HPLC-MS/MS. 1579 proteins were identified in this study which corresponds to 52% of the total proteome of *D. alaskensis* G20,[2] providing comprehensive coverage of the bacterial response to the presence of these two transition metals, relative to previous studies.[3,4]

After 2 h incubation there was a remarkable reduction in the protein abundance in treatments containing 100 ppm of Ni^{2+} in comparison to the control. It is also particularly noteworthy that metal-binding proteins, such as the UPF0173 metal-dependent hydrolase (Dde_0151), the quinone-interacting membrane-bound oxidoreductase (Dde_1113) and the MJ0042 family finger-like protein (Dde_1116), showed the highest abundance in the presence of 100 ppm Co^{2+} and yet the lowest abundance for the 100 ppm bimetallic treatment (Fig. S3B). UPF0173 metal-dependent hydrolases and MJ0042 family finger-like proteins are known for binding Zn^{2+} that can be exchanged by Co^{2+} without a loss of functionality. [5,6] To reduce the risk of mis-metallation with Ni, or perhaps to remedy such, it is understandable that a decrease in their abundance is observed and this might then also explain why Co removal from the dissolved fraction dropped when Ni^{2+} was present at concentrations ≥ 50 ppm.

After 20 h incubation, 23 proteins showed higher abundance in the treatment with Ni^{2+} compared to the Control and Co^{2+} treatments (Fig. S3C). Some of these proteins are likely responsible for mediating Ni^{2+} toxicity, such as the zinc resistance-associated protein (Dde_0111) which belongs to a family of four-helix hooked hairpins.[7] Also proteins that might be involved in metal reduction processes such as oxidoreductases,[8,9] were significantly more abundant after 20 h incubation with 10 ppm of Ni^{2+} compared to the treatment with Co^{2+}, confirming a distinctive cellular response depending on the metal. Some of these oxidoreductases, such as the FAD/NAD (P)-binding domain protein (Dde_1381) and the FAD-dependent pyridine nucleotide-disulfide oxidoreductase (Dde_2176), classified within the xenobiotics biodegradation and metabolism pathways, might be responsible for reducing Ni^{2+} into a less toxic form of this metal.

We identified similar cellular responses to Co^{2+}, and Ni^{2+}, with regard to ABC transporters as the periplasmic component of zinc ABC transporter protein (Dde_2208) and the Molybdenum ABC transporter (Dde_0155), were both more abundant in the presence of any metal treatment compared to the control. Dde_0155 had been identified in previous work with *D. alaskensis* G20 after incubation with Pd^{2+} and Pt^{4+} [1] suggesting that this protein is a key component of heavy metal detoxification pathways.[10] Conversely, other proteins related to the ABC transporter pathways were found at significantly larger concentrations after incubation with Co^{2+} alone than after treatments containing Ni^{2+}. These were two periplasmic subunit family 3 proteins, Dde_0168 (Fig. S3A-B) and Dde_1429 (Fig. S3A) related to export mechanisms and the cell division ATP-binding FtsE protein (Dde_0114) (Fig. S3A-B).[1]

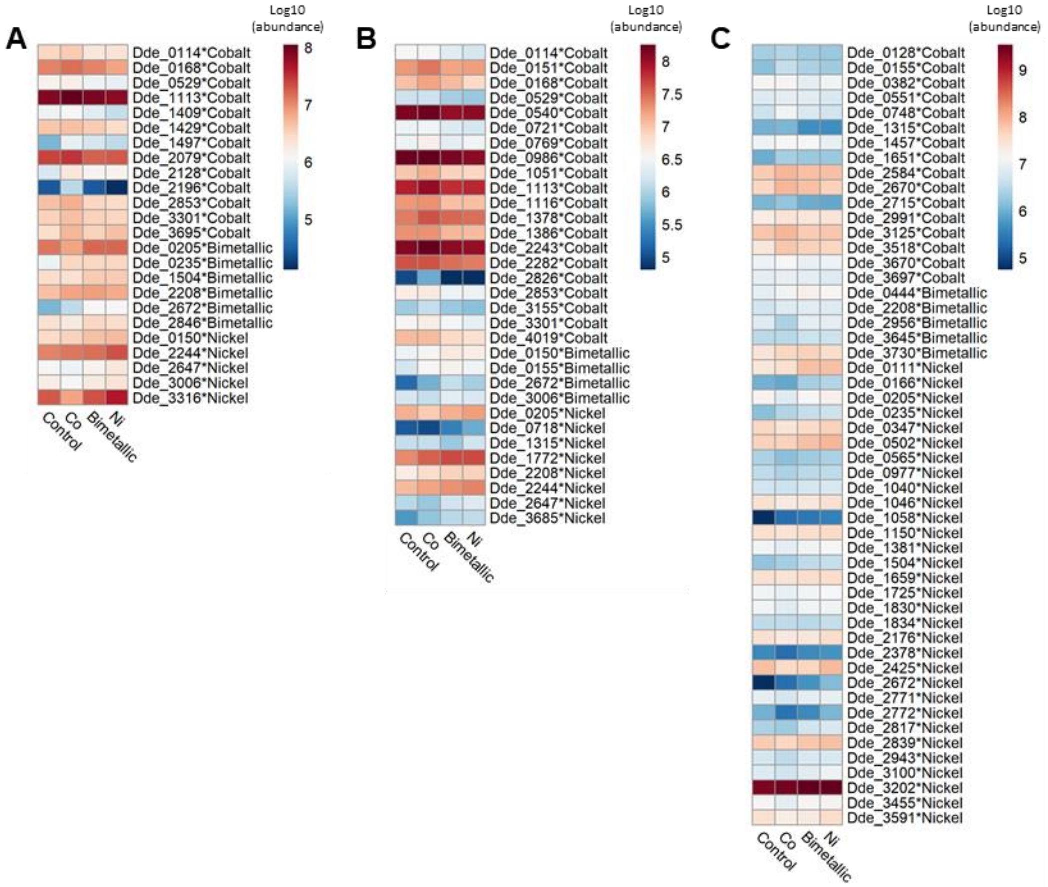

Fig. S3 Proteomics analysis. Heat maps of proteins abundance associated to the control and metal treatments (Co, Ni and bimetallic) after 2 h incubation with metals at (A) 10 ppm and (B) 100 ppm and (C) after 20 h incubation with metals at 10 ppm. Values are represented as the log 10 (Mean protein abundance, n=3 biological replicates). Rows are labelled with the protein identifier Dde_#### followed by the *metal treatment exhibiting the highest significant abundance (ANOVA, p-value <0.05, number of peptides detected ≥ 2, absolute ratio abundance > 1.5-fold). The protein abundance, ANOVA results and full name of the proteins together with their associated ID is available at https://doi.org/10.7488/ds/3130.

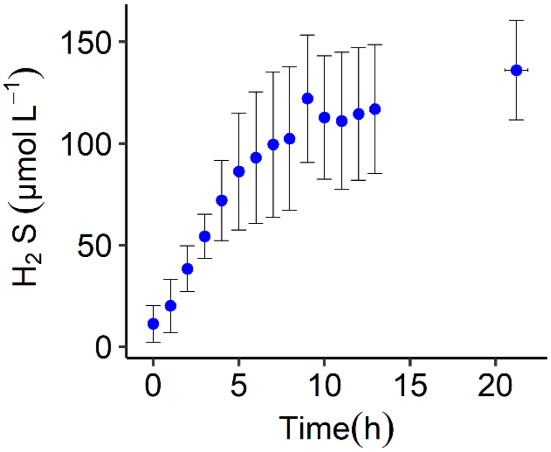

Fig. S4 Dissolved H_2S (Mean ± SD, n=3 biological replicates) measured over time in *D. alaskensis G20* cell cultures in the absence of metals (control treatment).

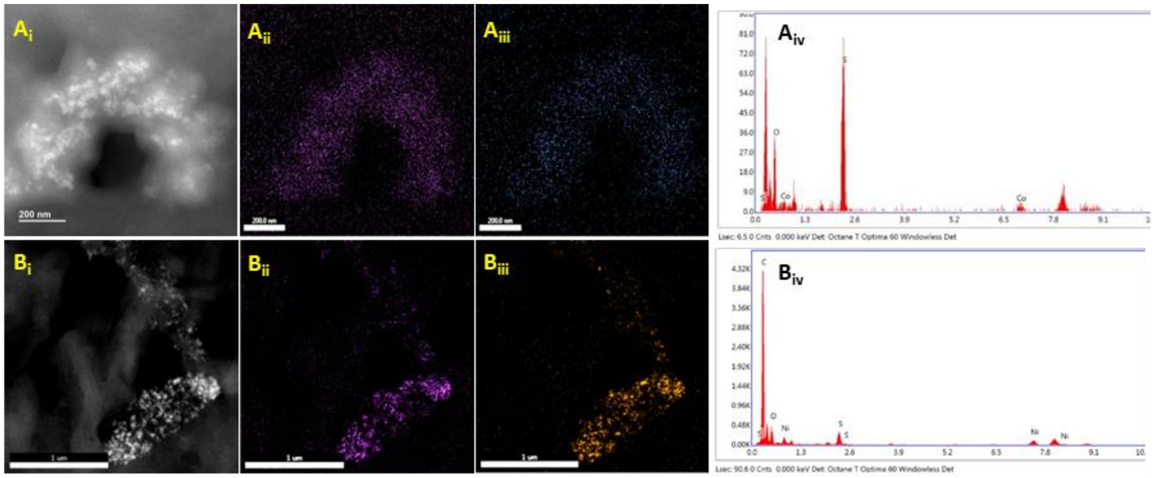

Fig. S5 STEM+EDX characterisation of *D. alaskensis* G20 incubated with Co^{2+} and Ni^{2+}. (A_i) STEM image of samples of *D. alaskensis* G20 incubated for 20 h in Co^{2+} 10 ppm, and re-suspended at a final concentration of EtOH 50 % v/v. (A_{ii}) EDXS elemental map for sulfur (A_{iii}) EDXS elemental map for Co and (A_{iv}) the related EDXS spectra, scale bar (A_i- A_{iii}) = 200 nm. (B_i) STEM image of samples of *D. alaskensis* G20 incubated for 20 h with Ni^{2+} 10 ppm, and re-suspended at a final concentration of EtOH 50 % v/v. (B_{ii}) EDXS elemental map for sulphur (B_{iii}) EDXS elemental map for Ni and (B_{iv}) the related EDXS spectra, scale bar (B_i- B_{iii}) = 1µm.

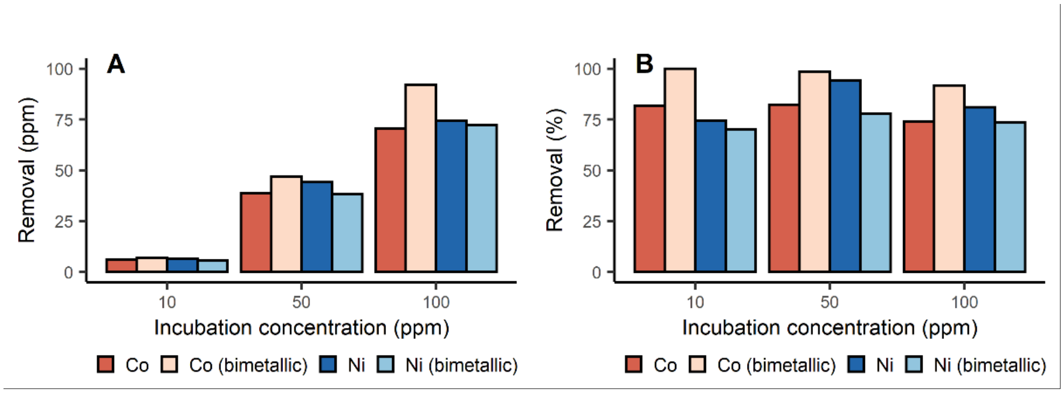

Fig. S6 Removal of Co^{2+} and Ni^{2+} measured as (A) ppm and (B) percentage with cell-free supernatant of *D. alaskensis G20* cultures, n=1.

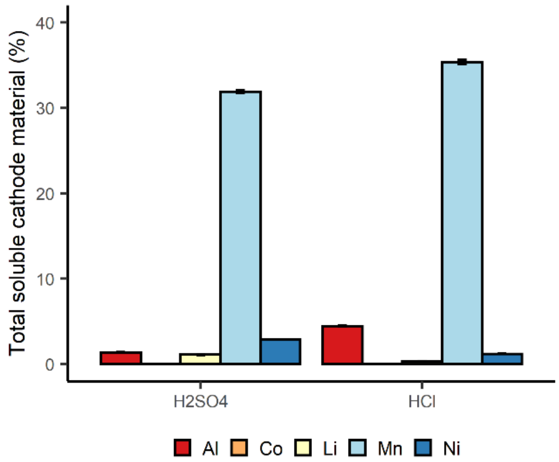

Fig. S7 Total soluble cathode leached (B1C) incubated for 5 h at 20 °C with 1 M of H_2SO_4 and HCl, expressed as the Mean (%) (n=2).

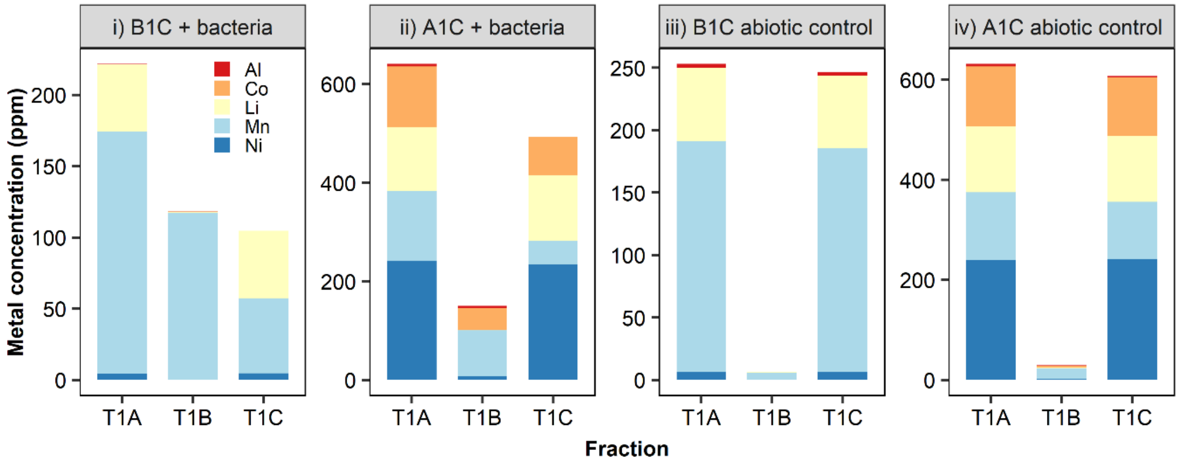

Fig. S8 Bioprecipitation of metals contained in vehicular LIBs using S. *oneidensis* MR-1. Leachates were obtained from B1C and A1C cathode material treated with 0.5 M H_2SO_4 at 50 °C using (i-ii) *S. oneidensis* MR-1 cell culture (mean value, n=3 biological replicates) and (iii-iv) LBNS medium as abiotic control (n=2 technical replicates).

Table S1. Dissolved metal concentration (ppm) in leachates incubated with *S. oneidensis* MR-1

Element	0.1M H_2SO_4; A1C (5' at 50 °C)	0.1M H_2SO_4; A1C (30' at 50 °C)	0.1M H_2SO_4; B1C (5' at 50 °C)	0.1M H_2SO_4; B1C (30' at 50 °C)	0.1M HCl; B1C (5' at 20 °C)	0.1M HCl; B1C (300' at 20 °C)
**Al**	1.16	5.01	3.56	11.2	12.2	42.9
**Co**	36.3	73.4	0.00	4.14	0.00	0.00
**Li**	41.6	77.9	32.0	64.0	5.99	17.2
**Mn**	47.2	83.6	108	206	45.0	119
**Ni**	67.5	143	4.55	39.5	0.00	0.07
**Total metal mass (ppm)**	194	383	148	325	63.1	179

Table S2. Metal removal from LIBs leachates after incubation with *S. oneidensis* MR-1

Leachate	Element	Removal (ppm)		Removal (%)		Precipitated
		Mean	SD	Mean	SD	Mass (%)
0.1 M H_2SO_4 A1C (5' at 50 °C)	Al	0.09	0.08	8.00	7.05	0.38
	Co	0.49	0.48	1.35	1.29	2.06
	Li	0.04	0.07	0.09	0.16	0.16
	Mn	23.24	3.89	49.36	9.12	97.20
	Ni	0.05	0.08	0.07	0.12	0.19
	Total	23.91		12.34		
0.1 M H_2SO_4 A1C (30' at 50 °C)	Al	2.69	0.80	53.96	16.44	5.37
	Co	6.72	1.30	9.20	2.02	13.39
	Li	0.04	0.06	0.05	0.08	0.07
	Mn	39.49	13.53	47.41	16.88	78.63
	Ni	1.28	1.77	0.87	1.19	2.54
	Total	50.22		13.12		
0.1 M H_2SO_4 B1C (5' at 50 °C)	Al	1.03	0.65	28.75	17.88	1.61
	Co	0.00	0.00	0.00	0.00	0.00
	Li	0.09	0.15	0.27	0.47	0.14
	Mn	62.75	27.69	57.94	25.01	98.25
	Ni	0.00	0.00	0.00	0.00	0.00
	Total	63.87		43.20		
0.1 M H_2SO_4 B1C (30' at 50 °C)	Al	7.83	1.56	69.87	12.01	4.70
	Co	0.86	0.15	20.82	3.02	0.52
	Li	2.98	3.07	4.65	4.87	1.79
	Mn	153.88	1.82	74.79	2.21	92.49
	Ni	0.83	0.95	2.06	2.34	0.50
	Total	166.38		51.24		
0.1 M HCl B1C (5' at 20 °C)	Al	8.98	0.45	74.53	10.88	30.40
	Co	0.00	0.00	0.00	0.00	0.00
	Li	0.01	0.02	0.19	0.34	0.04
	Mn	20.55	5.78	45.83	13.64	69.56
	Ni	0.00	0.00	0.00	0.00	0.00
	Total	29.54		46.78		
0.1 M HCl B1C (300' at 20 °C)	Al	39.84	0.79	92.99	3.81	32.10
	Co	0.00	0.00	0.00	0.00	0.00
	Li	0.26	0.17	1.50	0.94	0.21
	Mn	84.02	6.37	70.87	5.45	67.69
	Ni	0.01	0.01	3.59	6.23	0.01
	Total	124.13		69.48		

n=3 biological replicates

Table S3. Metal concentration (ppm) in the different fractions during T1 and T2 of leachate B1C 0.5 M H_2SO_4

	Data associated to Fig. 8A-i			
Element	T1A	T1B	T1C	Removal (%)
Al	9.88	3.15	6.74	31.8
Co	4.22	0.00	4.29	0.00
Li	79.2	0.0	79.4	0.00
Mn	207	186	21.1	89.8
Ni	42.5	0.00	43.0	0.00

	Data associated to Fig. 8A-ii 25 % v/v leachate								
Element	Cell-only (n=3)						MOPS (n=1)		
	T2A		T2B		T2C		T2A	T2B	T2C
	Mean	SD	Mean	SD	Mean	SD			
Al	0.02	0.04	0.02	0.04	0.00	0.00	0.00	0.00	0.00
Co	0.34	0.07	0.34	0.07	0.00	0.00	0.44	0.00	0.48
Li	18.6	0.30	0.13	0.22	18.7	0.10	19.0	0.00	19.0
Mn	3.11	0.15	3.11	0.15	0.00	0.00	3.08	3.08	0.00
Ni	9.40	0.28	2.46	0.41	6.94	0.62	10.0	0.00	10.4

	Data associated to Fig. 8A-iii 50 % v/v leachate								
Element	Cell-only (n=3)						MOPS (n=1)		
	T2A		T2B		T2C		T2A	T2B	T2C
	Mean	SD	Mean	SD	Mean	SD			
Al	1.69	0.12	1.02	0.17	0.67	0.11	1.90	1.00	0.90
Co	1.68	0.07	1.51	0.16	0.17	0.19	1.89	0.02	1.87
Li	37.3	0.30	0.18	0.16	37.3	0.70	38.1	0.37	37.7
Mn	8.21	0.22	8.21	0.22	0.00	0.00	8.64	8.64	0.00
Ni	20.1	0.1	4.86	1.28	15.2	1.30	21.7	0.08	21.6

	Data associated to Fig. 8A-iv 50 % v/v leachate								
Element	Whole culture (n=3)						Media (n=1)		
	T2A		T2B		T2C		T2A	T2B	T2C
	Mean	SD	Mean	SD	Mean	SD			
Al	1.78	0.04	0.87	0.18	0.91	0.14	1.71	0.43	1.28
Co	1.54	0.04	1.43	0.01	0.11	0.04	1.86	0.00	1.96
Li	37.7	0.80	0.22	0.38	37.8	0.30	37.2	0.00	37.7
Mn	8.79	1.30	8.79	1.30	0.00	0.00	9.19	6.47	2.72
Ni	19.4	0.60	7.59	0.93	11.8	0.40	21.2	0.00	21.7

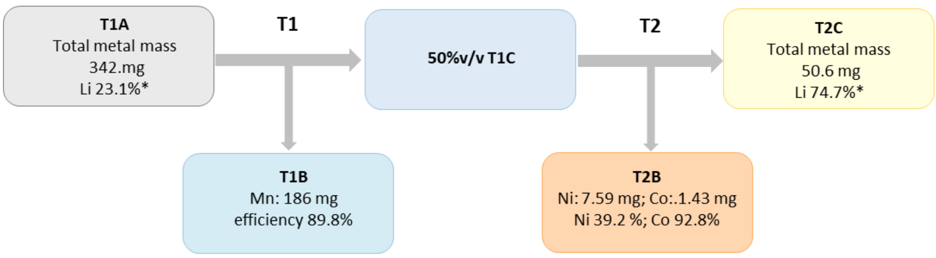

Fig.S9 Mass flow of bioprocess with B1C leachate0.5 M H2SO4 (10%v/v leachate + 90 v/v bacterial culture) associated to Fig. 8 A i) B1C-0.5M - iv) 50% v/v (T1C). * % (mass Li/ total metal mass)

Table S4. Metal concentration (ppm) in the different fractions during T1 and T2 of leachate A1C 0.5 M H_2SO_4

	Data associated to Fig. 8B-i			
**Element**	**T1A**	**T1B**	**T1C**	**Removal (%)**
Al	10.8	10.8	0.00	100
Co	142	53.5	88.2	38
Li	154	1.00	153	1
Mn	160	155	5.10	97
Ni	286	25.1	261	9

	Data associated to Fig. 8B-ii 25 % v/v leachate								
**Element**	**Cell-only (n=3)**						**MOPS (n=1)**		
	**T2A**		**T2B**		**T2C**		**T2A**	**T2B**	**T2C**
	**Mean**	**SD**	**Mean**	**SD**	**Mean**	**SD**			
Al	0.00	0.00	0.00	0.00	0.00	0.00	0.00	0.00	0.00
Co	25.6	0.28	4.28	0.99	21.4	0.76	25.6	0.00	26.1
Li	40.1	0.37	0.00	0.00	40.8	0.14	39.9	0.00	40.6
Mn	0.11	0.03	0.11	0.03	0.00	0.00	0.23	0.14	0.09
Ni	68.0	1.42	12.4	3.12	55.6	1.71	68.7	0.00	69.3

	Data associated to Fig. 8B-iii 50 % v/v leachate								
**Element**	**Cell-only (n=3)**						**MOPS (n=1)**		
	**T2A**		**T2B**		**T2C**		**T2A**	**T2B**	**T2C**
	**Mean**	**SD**	**Mean**	**SD**	**Mean**	**SD**			
Al	0.00	0.00	0.00	0.00	0.00	0.00	0.00	0.00	0.00
Co	49.9	0.41	4.36	0.23	45.5	0.64	50.1	0.00	51.5
Li	81.2	0.66	0.00	0.00	81.6	0.7	81.4	0.00	82.7
Mn	2.07	0.02	0.38	0.04	1.69	0.05	2.06	0.00	2.12
Ni	139	1.05	11.5	1.48	127	1.15	140	0.00	145

	Data associated to Fig. 8B-iv 50 % v/v leachate								
**Element**	**Whole culture (n=3)**						**Media (n=1)**		
	**T2A**		**T2B**		**T2C**		**T2A**	**T2B**	**T2C**
	**Mean**	**SD**	**Mean**	**SD**	**Mean**	**SD**			
Al	0.00	0.00	0.00	0.00	0.00	0.00	0.00	0.00	0.00
Co	48.4	0.31	48.4	0.31	0.00	0.00	48.6	0.00	50.1
Li	80.9	0.44	0.45	0.78	81.1	1.16	81.5	0.00	82.4
Mn	1.96	0.02	0.14	0.04	1.83	0.05	1.94	0.00	2.02
Ni	138	0.38	133	2.32	5.39	2.12	139	0.00	143

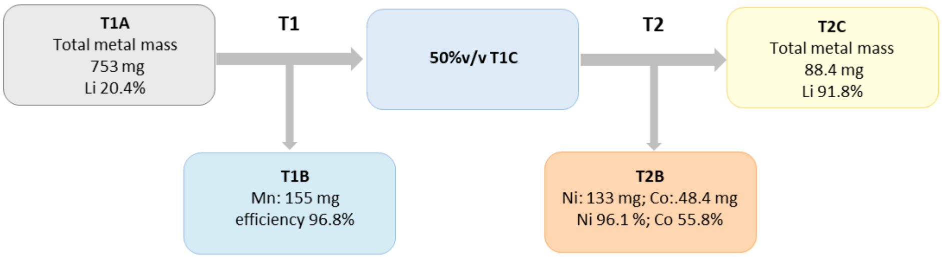

Fig.S10 Mass flow of bioprocess with A1C leachate0.5 M H2SO4 (10%v/v leachate + 90 v/v bacterial culture) associated to Fig. 8 B i) A1C-0.5M - iv) 50% v/v (T1C). * % (mass Li/ total metal mass)

Table S5. Metal concentration (ppm) in the different fractions during T1 and T2 of leachate A1C 0.1 M H_2SO_4

	Data associated to Fig. 8C-i			
Element	T1A	T1B	T1C	Removal (%)
Al	0.00	0.00	0.00	0.00
Co	50.7	0.46	50.2	5.40
Li	59.3	0.00	59.9	1.50
Mn	57.9	51.5	6.39	89.7
Ni	94.8	0.00	96.1	2.80

	Data associated to Fig. 8C-ii 25 % v/v leachate								
Element	Cell-only (n=3)						MOPS (n=1)		
	T2A		T2B		T2C		T2A	T2B	T2C
	Mean	SD	Mean	SD	Mean	SD			
Al	0.00	0.00	0.00	0.00	0.00	0.00	0.00	0.00	0.00
Co	11.3	0.07	7.17	0.30	4.17	0.37	12.2	0.00	12.4
Li	14.5	0.10	0.09	0.15	14.4	0.20	14.5	0.00	14.6
Mn	0.00	0.00	0.00	0.00	0.00	0.00	0.00	0.00	0.00
Ni	22.3	0.35	5.68	0.68	16.6	0.33	23.6	0.00	23.6

	Data associated to Fig. 8C-iii 50 % v/v leachate								
Element	Cell-only (n=3)						MOPS (n=1)		
	T2A		T2B		T2C		T2A	T2B	T2C
	Mean	SD	Mean	SD	Mean	SD			
Al	0.00	0.00	0.00	0.00	0.00	0.00	0.00	0.00	0.00
Co	24.1	0.75	12.20	2.63	11.9	2.13	26.1	0.29	25.8
Li	27.7	0.82	0.07	0.06	27.8	0.52	28.9	0.35	28.5
Mn	0.89	0.06	0.74	0.06	0.15	0.06	0.97	0.31	0.66
Ni	46.1	1.59	7.91	2.12	38.2	1.50	49.3	0.53	48.8

	Data associated to Fig. 8C-iv 50 % v/v leachate								
Element	Whole culture (n=3)						Media (n=1)		
	T2A		T2B		T2C		T2A	T2B	T2C
	Mean	SD	Mean	SD	Mean	SD			
Al	0.00	0.00	0.00	0.00	0.00	0.00	0.00	0.00	0.00
Co	24.0	0.04	20.50	0.33	3.50	0.33	24.6	0.00	25.1
Li	28.3	0.10	0.00	0.00	28.8	0.08	27.5	0.00	28.2
Mn	0.88	0.05	0.23	0.02	0.65	0.02	1.63	0.14	1.49
Ni	46.4	0.09	23.80	1.92	22.5	1.97	48.6	0.38	48.2

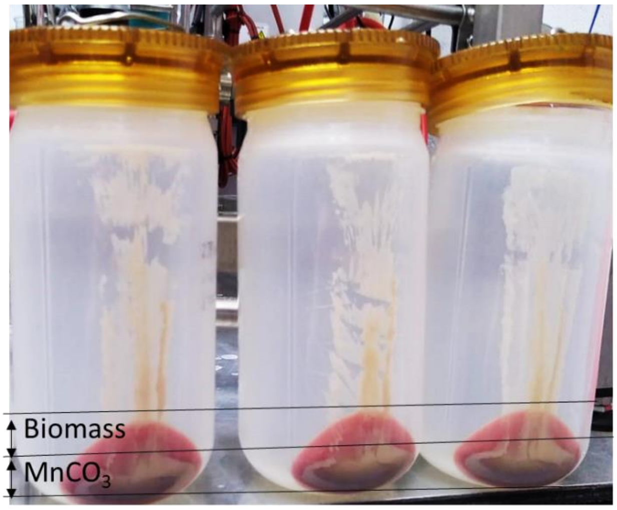

Fig. S11 Bioprecipiated Mn as $MnCO_3$ (beige layer) below the biomass layer (pink) after centrifugation to separate the solid and dissolved fractions.

References

1 M. J. Capeness, L. Imrie, L. F. Mühlbauer, T. Le Bihan and L. E. Horsfall, *Microbiology*, 2019, **165**, 1282–1294.

2 L. J. Hauser, M. L. Land, S. D. Brown, F. Larimer, K. L. Keller, B. J. Rapp-Giles, M. N. Price, M. Lin, D. C. Bruce, J. C. Detter, R. Tapia, C. S. Han, L. A. Goodwin, J.-F. Cheng, S. Pitluck, A. Copeland, S. Lucas, M. Nolan, A. L. Lapidus, A. V Palumbo and J. D. Wall, *J. Bacteriol.*, 2011, **193**, 4268–4269.

3 Y. Liu, A. Serrano, V. Wyman, E. Marcellin, G. Southam, J. Vaughan and D. Villa-Gomez, *J. Hazard. Mater.*, 2021, **402**, 123506.

4 H. Dulay, M. Tabares, K. Kashefi and G. Reguera, *Front. Microbiol.*, 2020, **11**, 2992.

5 W. M. B. L. Vallee, *Methods Enzymol.*, 1993, **226**, 52–71.

6 B. Bennett, in *Metals in Biology. Biological Magnetic Resonance, vol 29.*, eds. G. Hanson and L. Berliner, Springer New York, New York, NY, 2010, pp. 345–370.

7 T. Schmidt and H. G. Schlegel, *J. Bacteriol.*, 1994, **176**, 7045–7054.

8 A. E. Otwell, R. W. Sherwood, S. Zhang, O. D. Nelson, Z. Li, H. Lin, S. J. Callister and R. E. Richardson, *Environ. Microbiol.*, 2015, **17**, 1977–1990.

9 M. Arenas-Salinas, J. I. Vargas-Pérez, W. Morales, C. Pinto, P. Muñoz-Díaz, F. A. Cornejo, B. Pugin, J. M. Sandoval, W. A. Díaz-Vásquez, C. Muñoz-Villagrán, F. Rodríguez-Rojas, E. H. Morales, C. C. Vásquez and F. A. Arenas, *Front. Microbiol.*, 2016, **7**, 1160.

10 J. Y. Lee, J. G. Yang, D. Zhitnitsky, O. Lewinson and D. C. Rees, *Science.*, 2014, **343**, 1133–1136.
